# GCN5-dependent acetylation of HIV-1 integrase enhances viral integration

**DOI:** 10.1186/1742-4690-7-18

**Published:** 2010-03-12

**Authors:** Mariaelena Terreni, Paola Valentini, Vania Liverani, Maria Ines Gutierrez, Cristina Di Primio, Armida Di Fenza, Valentina Tozzini, Awatef Allouch, Alberto Albanese, Mauro Giacca, Anna Cereseto

**Affiliations:** 1 Molecular Biology Laboratory, Scuola Normale Superiore, Piazza dei Cavalieri 7, 56100 Pisa, Italy; 2 Molecular Medicine Laboratory, International Centre for Genetic Engineering and Biotechnology (ICGEB), Padriciano 99, 34012 Trieste, Italy; 3 NEST, Istituto Nanoscienze - CNR and Scuola Normale Superiore, Piazza San Silvestro 12, 56127, Pisa, Italy

## Abstract

**Background:**

An essential event during the replication cycle of HIV-1 is the integration of the reverse transcribed viral DNA into the host cellular genome. Our former report revealed that HIV-1 integrase (IN), the enzyme that catalyzes the integration reaction, is positively regulated by acetylation mediated by the histone acetyltransferase (HAT) p300.

**Results:**

In this study we demonstrate that another cellular HAT, GCN5, acetylates IN leading to enhanced 3'-end processing and strand transfer activities. GCN5 participates in the integration step of HIV-1 replication cycle as demonstrated by the reduced infectivity, due to inefficient provirus formation, in GCN5 knockdown cells. Within the C-terminal domain of IN, four lysines (K258, K264, K266, and K273) are targeted by GCN5 acetylation, three of which (K264, K266, and K273) are also modified by p300. Replication analysis of HIV-1 clones carrying substitutions at the IN lysines acetylated by both GCN5 and p300, or exclusively by GCN5, demonstrated that these residues are required for efficient viral integration. In addition, a comparative analysis of the replication efficiencies of the IN triple- and quadruple-mutant viruses revealed that even though the lysines targeted by both GCN5 and p300 are required for efficient virus integration, the residue exclusively modified by GCN5 (K258) does not affect this process.

**Conclusions:**

The results presented here further demonstrate the relevance of IN post-translational modification by acetylation, which results from the catalytic activities of multiple HATs during the viral replication cycle. Finally, this study contributes to clarifying the recent debate raised on the role of IN acetylated lysines during HIV-1 infection.

## Background

Integration of reverse transcribed HIV-1 DNA into the cellular genome is catalyzed by the viral IN protein. Even though *in vitro *integration can be solely driven by IN, cellular cofactors are required to complete the reaction *in vivo*. It was recently reported that the cellular HAT p300 interacts with IN and regulates its function through acetylation [1,2]. HATs are enzymes able to transfer acetyl groups from acetyl coenzyme A (acetyl-CoA) to specific lysine residues within the N-terminal tails of nucleosomal histones, leading to chromatin decondensation and transcriptional activation [3,4]. HATs can also acetylate non-histone substrates, such as transcription factors and other nuclear proteins, as well as cytoskeletal components, metabolic enzymes and signalling regulators in the cytoplasm [5]. Acetylation has been reported to regulate the activity of these factors by modulating DNA binding [6-8], protein-protein interactions [9-12], protein stability [13-15], and subcellular localization [16-19]. Growing evidence now indicates that acetylation significantly participates in signaling pathways ultimately regulating viral infectivity [20-26]. Among the viral factors functionally modulated by acetylation is the HIV-1 protein Tat. Tat is acetylated at lysine 28 by PCAF, while residues 50 and 51 are substrates for p300/CBP and GCN5 [27-29]. Acetylation of lysine 28 enhances the ability of Tat to recruit the P-TEFb complex [28], while modification of lysine 50 leads to Tat dissociation from TAR RNA [28,30]. Therefore, even though the final effect of acetylation is an increased transactivation activity on the viral LTR promoter, the modification of each individual lysine differently affects Tat functionality at the molecular level.

We have recently discovered that another HIV-1-encoded protein, IN, is a substrate for p300-mediated acetylation. Three lysine residues, located at positions 264, 266, and 273 in the C-terminal domain of IN, were identified as the target sites for modification [1,2]. Acetylation by p300 was shown to increase both IN affinity for DNA and strand transfer activity [1], thus suggesting a potential role for this post-translational modification during viral integration. The importance of IN acetylation for HIV-1 replication was further highlighted by the finding that the mutant virus, in which arginine substitutions were introduced at p300-targeted IN lysines, integrated less efficiently than the wild type [1].

Since proteins modified by acetylation are often substrates for multiple HATs, we sought to investigate whether IN might be acetylated by enzymes other than p300. It has already been reported that MOZ and PCAF (belonging to the MYST and GNAT families of HATs, respectively) are incapable of efficiently acetylating the IN C-terminal domain *in vitro *[2]. Therefore, in this study, another member of the GNAT family, GCN5, was examined. Here we demonstrate that GCN5 binds and acetylates IN both *in vitro *and *in vivo*. GCN5 expression is functionally relevant to HIV-1 infectivity and specifically affects the integration process, likely by modulating the catalytic activity of IN. Interestingly, the four lysines targeted by GCN5 partially overlap with those modified by p300 in the C-terminal domain of IN. A comparative analysis of viral clones mutated at IN lysines acetylated by GCN5 or p300 revealed the same replication defect at the step of integration, thus indicating common roles for the two HATs in regulating IN function.

## Results

### HIV-1 IN is acetylated by GCN5

**Figure 1:**
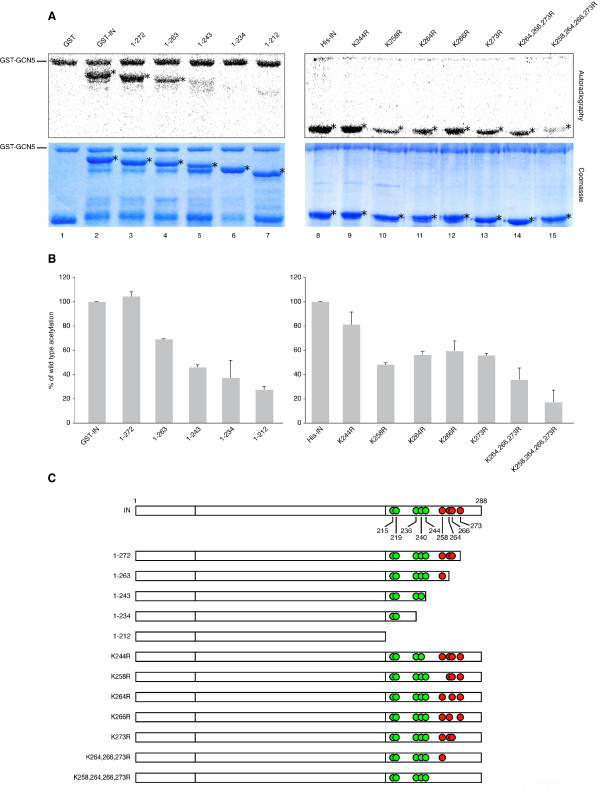
**HIV-1 IN is acetylated by GCN5 *in vitro***. (A) Autoradiography (upper panels) and Coomassie blue staining (lower panels) of *in vitro *acetylation assay with recombinant GST-GCN5 and IN wild type or mutant proteins. Lanes 1-7: GST fusion IN proteins; lanes 8-15: 6× His-tagged IN proteins. In the Coomassie panels, IN proteins used as acetylation substrates are indicated by asterisks; in the autoradiograms, IN proteins found positive for GCN5-mediated acetylation are indicated in the same way. Presented results are representative data from triplicate *in vitro *acetylation assay experiments. (B) Results of densitometric analysis of autoradiograms derived from three independent experiments (means ± standard errors of the means [SEM]) expressed as percent wild type IN acetylation. (C) Schematic representation of IN proteins used for the acetylation assays. The positions of lysines in the C-terminal domain of IN are indicated. Lysines positive for acetylation are shown in red.

To examine whether IN is acetylated by GCN5, *in vitro *acetylation assays were performed with recombinant IN and GCN5, both purified as GST fusion proteins. Incubation of the single GST domain with GCN5 in the presence of [^14^C]-acetyl-CoA, and subsequent protein resolution by SDS-PAGE followed by autoradiography, revealed a unique band at the same size as GST-GCN5, corresponding to the auto-acetylation product of the enzyme (Figure 1A, lane 1). Incubation of GST-IN with GST-GCN5 resulted in two major radiolabeled bands, the higher one corresponding to auto-acetylated GST-GCN5 and the lower one to GST-IN (Figure 1A, lane 2), thus demonstrating that GCN5 specifically acetylates IN *in vitro*.

To define which region of IN is acetylated by GCN5, GST-IN fragments with progressive deletions starting from the C-terminus (as schematized in Figure 1C) were used as substrates in *in vitro *acetylation assays, and the corresponding acetylation signals in the autoradiograms were evaluated by densitometric analysis (Figure 1B, left histogram). GST-IN fragment 1-272 was acetylated to a similar extent as full-length IN (Figure 1A, compare lanes 2 and 3, and Figure 1B, left histogram). Acetylation of fragment 1-263 (Figure 1A, lane 4) was reduced by 30% (Figure 1B, left histogram), while a more significant decrease in the signal (ranging from 60% to 70%) was observed using shorter fragments (1-243, 1-234 and 1-212) (Figure 1A, lanes 5-7, and Figure 1B, left histogram). These results indicated that IN is acetylated by GCN5 within the region located between amino acids 244 and 288. As schematically represented in Figure 1C, this region contains five lysines at positions 244, 258, 264, 266, and 273 as possible targets for acetylation. Therefore, in order to exclude that the reduced acetylation of the deleted IN forms resulted from improper protein folding, each of these lysines was replaced with an arginine, an amino acid that cannot be acetylated and conserves a positively charged side chain. The resulting mutants were then tested *in vitro *as substrates for GCN5 activity. In this experiment, IN was tagged with a 6× His epitope in place of GST, in order to obtain better SDS-PAGE resolution between acetylated GCN5 and IN (Figure 1A, lane 8). As reported in the right histogram of Figure 1B, densitometric analysis of radioactivity incorporation highlighted that the mutation of the individual lysines 258, 264, 266, and 273 (Figure 1A, lanes 10-13) caused a reduction in the acetylation level of IN ranging from 40% to 50%, while no significant decrease in the signal was detected upon mutation of lysine 244 (Figure 1A, lane 9). These data suggested that GCN5 acetylates IN at residues 258, 264, 266, and 273. Notably, previous reports demonstrated that another HAT, p300, acetylates lysines 264, 266, and 273 of IN [1,2]. To confirm that GCN5 acetylates lysine 258 in addition to the above-mentioned residues, two mutant forms of IN were assayed for *in vitro *acetylation: one containing mutations at the sites acetylated by both GCN5 and p300 (IN K264,266,273R), and the other carrying these same amino acidic substitutions, with the additional mutation of lysine 258 specifically targeted by GCN5 (IN K258,264,266,273R). The decrease in the radioactive signal detected with IN K264,266,273R was similar to the one obtained with the single-mutated forms (compare lane 14 with lanes 10-13 in Figure 1A, and right histogram of Figure 1B), while the residual acetylation level of IN K258,264,266,273R dropped to 20% with respect to wild type (Figure 1A, lane 15, and Figure 1B, right histogram). These results demonstrated that GCN5 acetylates lysines 264, 266, and 273 of IN, also targeted by p300, and lysine 258 as a specific site of modification.

**Figure 2:**
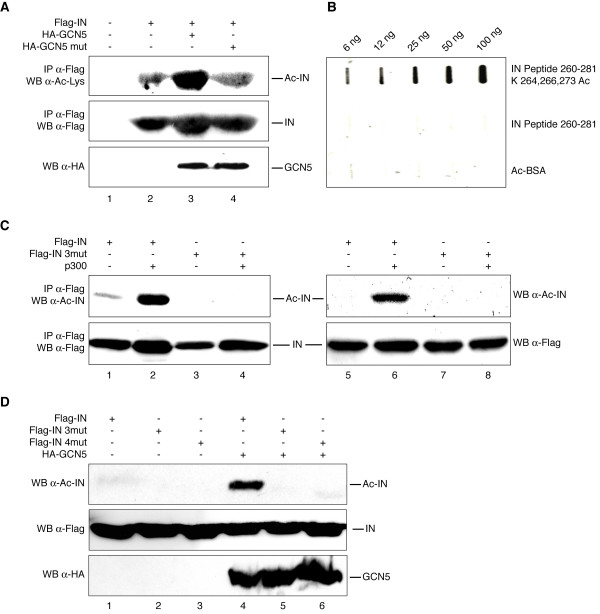
**IN is acetylated by GCN5 *in vivo***. (A) Extracts from HEK 293T cells transfected with the indicated plasmids were immunoprecipitated using anti-Flag antibody and analyzed by Western blotting with anti-acetyl-lysine antibody (upper panel) or anti-Flag antibody (middle panel). Lower panel: cell extracts immunoblotted with anti-HA antibody. (B) Acetylated BSA and peptides corresponding to IN amino acids 260-281, either chemically acetylated at lysines 264, 266, and 273, or not acetylated, were blotted onto a nitrocellulose filter and incubated with anti-acetylated IN antibody. (C) Left panels (lanes 1-4): extracts from HEK 293T cells transfected with the indicated plasmids were immunoprecipitated using anti-Flag antibody and analyzed by Western blotting with anti-acetylated IN antibody (top panel) or anti-Flag antibody (bottom panel). Right panels (lanes 5-8): extracts from HEK 293T cells transfected with the indicated plasmids analyzed by Western blotting with anti-acetylated-IN antibody (top panel) or anti-Flag antibody (bottom panel). (D) Extracts from HEK 293T cells transfected with the indicated plasmids analyzed by Western blotting with anti-acetylated-IN antibody (upper panel), anti-Flag antibody (middle panel), or anti-HA antibody (lower panel).

Next, we investigated whether IN is also acetylated by GCN5 *in vivo*. Codon-optimized Flag-IN [31] was expressed in HEK 293T cells, alone or together with HA-GCN5 wild type or mutated in the catalytic domain (Y260A/F261A) [32]. Immunoprecipitation of IN and subsequent detection by Western blotting with an antibody specific to acetylated lysines revealed the highest acetylation signal in the sample corresponding to IN co-expressed with wild type GCN5 (Figure 2A, upper panel, lane 3). Conversely, expression of IN alone or together with catalytically inactive GCN5 resulted in a lower acetylation signal, likely derived from the activity of endogenous HATs (Figure 2A, upper panel, lanes 2 and 4). In this experiment, the total amounts of immunoprecipitated IN and the expression levels of wild type and mutant GCN5 were verified by Western blot analysis with anti-Flag and anti-HA antibodies, respectively (Figure 2A, middle and lower panels).

### Detection of *in vivo *IN acetylation by a novel anti-acetylated IN antibody

To confirm the *in vitro *observation that IN is a substrate for both GCN5 and p300, an antibody specific to acetylated IN was produced by using an IN-derived peptide for immunization. The IN-derived peptide was chemically acetylated at lysines 264, 266, and 273, which are targeted in common by the two HATs (see the Methods section). As shown in Figure 2B, the purified antibody specifically recognized the acetylated IN peptide in dot blot experiments, while no cross-reactivity was detected with the unmodified peptide or acetylated BSA. This antibody allowed detecting basal levels of IN acetylation by endogenous HATs following immunoprecipitation (Figure 2C, top-left panel, lane 1); additionally, high levels of IN acetylation were detected from cells overexpressing p300 (Figure 2C, top-left panel, lane 2). This result is consistent with our previous study showing that p300 mediates IN acetylation *in vivo *at positions 264, 266, and 273 [1]. Conversely, no signal, expressed either alone or together with p300 (Figure 2C, top-left panel, lanes 3 and 4), was detected with IN K264,266,273R, thus revealing the high specificity of the antibody. In this experiment, the amount of IN (wild type or mutated) immunoprecipitated in each sample was verified by Western blotting with an anti-Flag antibody (Figure 2C, bottom-left panel). The anti-acetylated IN antibody was also used for direct Western blot analysis of cell lysates, producing a strong acetylation signal in the sample corresponding to IN co-expressed with p300 (Figure 2C, top-right panel, lane 6). Therefore, the newly developed antibody showed higher sensitivity than the standard anti-acetyl-lysine antibodies, which require an immunoprecipitation step to reveal IN acetylation. Given the high specificity and sensitivity of the anti-acetylated IN antibody, it was used to confirm the *in vivo *acetylation of IN by GCN5, as well as the mapping of the *in vitro *targeted lysines. As shown in the upper panel of Figure 2D, extracts from cells co-expressing wild type IN and GCN5 revealed a remarkable signal corresponding to IN acetylation (lane 4); while, consistent with the data reported in Figure 2C (top right panel, lane 5), acetylation of the viral enzyme by endogenous HATs was almost undetectable (lane 1). Conversely, no signal with triple- and quadruple-mutant IN, expressed either alone (lanes 2 and 3) or together with GCN5 (lanes 5 and 6) was detected. In this experiment, Western blot analysis of the cell lysates was also performed with anti-Flag and anti-HA antibodies to control the levels of exogenously expressed proteins (Figure 2D, middle and lower panels). Taken together, these data demonstrated that IN is acetylated by GCN5 both *in vitro *and *in vivo*, and the targeted lysines are located in the C-terminal domain at positions 258, 264, 266, and 273.

### IN interacts with GCN5

**Figure 3:**
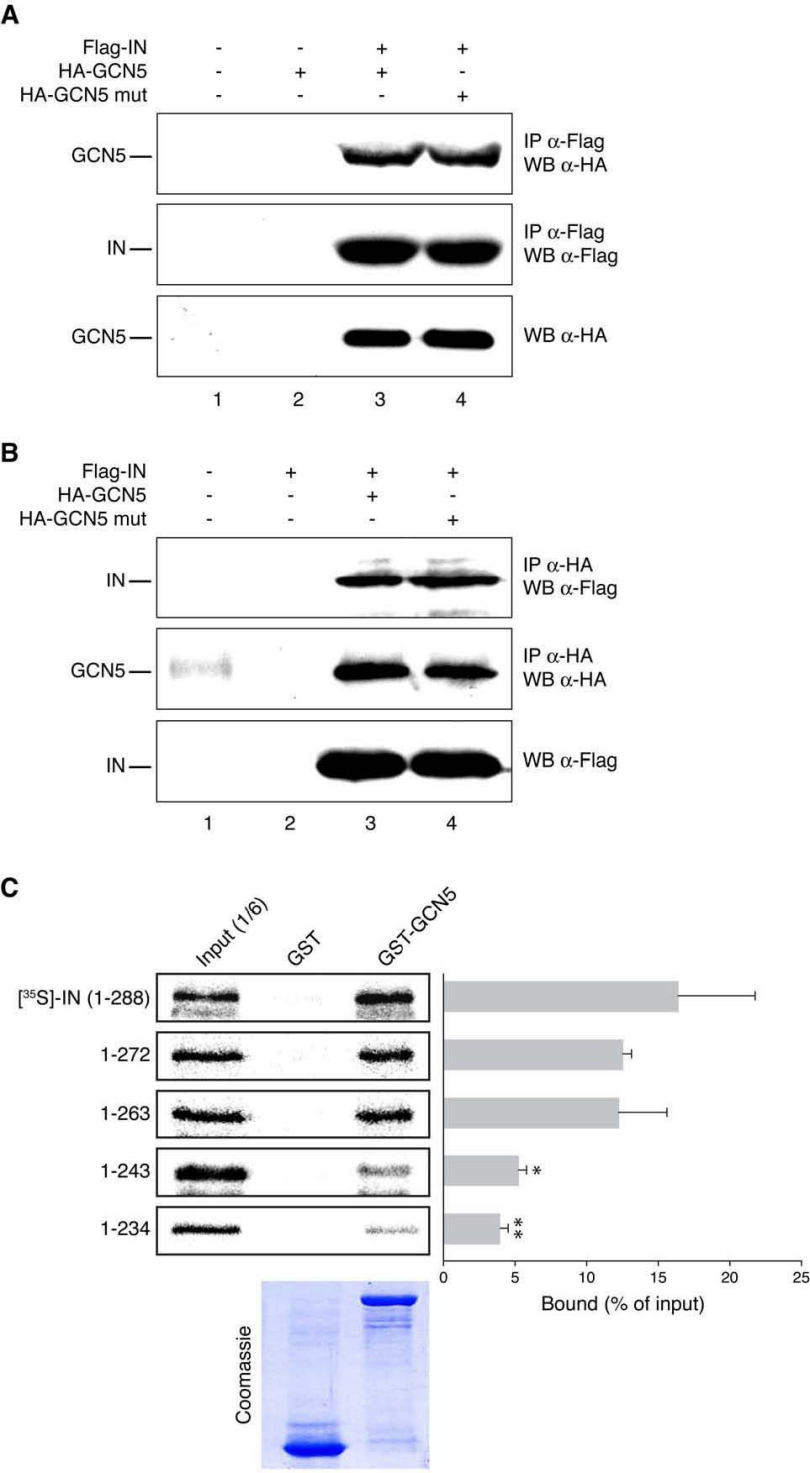
**IN interacts with GCN5 both *in vitro *and *in vivo***. (A) Extracts from HEK 293T cells transfected with the indicated plasmids were immunoprecipitated using anti-Flag antibody and analyzed by Western blotting with anti-HA antibody (upper panel) or anti-Flag antibody (middle panel). Lower panel: extracts immunoblotted with anti-HA antibody. (B) Extracts from HEK 293T cells transfected with the indicated plasmids were immunoprecipitated using anti-HA antibody and analyzed by Western blotting with anti-Flag antibody (upper panel) or anti-HA antibody (middle panel). Lower panel: extracts immunoblotted with anti-Flag antibody. (C) Autoradiography and Coomassie Blue staining of *in vitro *binding assays with GST-GCN5 and ^35^S-IN or the indicated ^35^S-IN fragments. The histogram represents the results of three independent experiments (means ± SEM), where the amounts of bound proteins are expressed as percentages of the corresponding radiolabeled inputs. Statistical significance of the binding percentages was calculated by using the Student's two-sided *t *test. Asterisks directly above bars indicate differences in binding efficiency to GST-GCN5 between IN deleted forms and full-length IN. **, *P *< 0,01; *, *P *< 0,05. Conversely, where asterisks are not present, values obtained did not significantly differ (*P *> 0,05) from those obtained with control, non-silenced cells.

Since IN is acetylated by GCN5, the interaction between these two factors was investigated. To this aim, HEK 293T cells were transfected with Flag-IN together with HA-GCN5 wild type or mutated in the catalytic domain. After immunoprecipitation with an anti-Flag antibody, both wild type and mutant GCN5 were found to co-precipitate with IN, as demonstrated by Western blot analysis using an anti-HA antibody (Figure 3A, upper panel, lanes 3 and 4). Accordingly, in the reciprocal experiment, where immunoprecipitation was performed with an anti-HA antibody, IN was found to associate with GCN5 (both wild-type and mutant forms) (Figure 3B, upper panel, lanes 3 and 4). In both experiments, the total amounts of immunoprecipitated proteins and the expression levels of IN and GCN5 were verified by Western blotting (Figures 3A and 3B, middle and lower panels).

To map the region of IN mediating the interaction with GCN5, pull-down assays were carried out between GST-GCN5 immobilized on glutathione-Sepharose beads and IN deletion mutants labeled with [^35^S]-Met by *in vitro *translation. As shown in Figure 3C, the affinities of IN fragments 1-272 and 1-263 to GST-GCN5 (13% binding efficiency) were similar to that of full-length IN (16% binding efficiency). Conversely, the GCN5/IN interaction significantly decreased using fragments containing further deletions towards the N-terminus (1-243 and 1-234). These results indicated that the C-terminal region of IN located between amino acids 244 and 288 is involved in binding to GCN5.

### Acetylation by GCN5 increases IN catalytic activity *in vitro*

**Figure 4:**
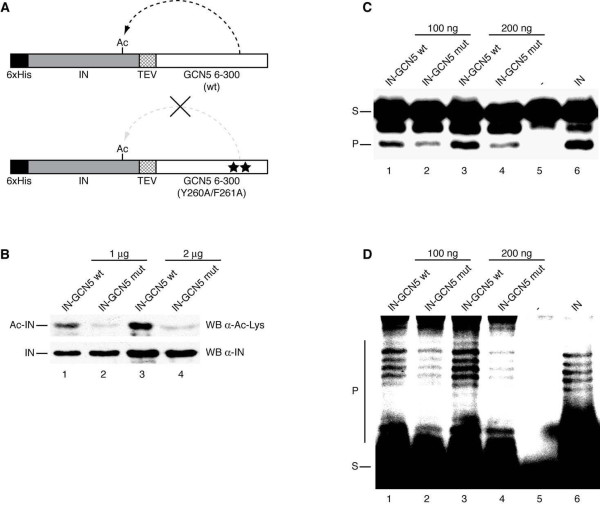
**GCN5-mediated acetylation increases the catalytic activity of IN**. (A) Schematic representation of IN-GCN5 tethered catalysis constructs. Full-length IN, tagged with a N-terminal 6× His epitope, is fused in frame with TEV proteolytic site and cloned upstream of the 6-300 amino acid region of wild type GCN5 (IN-HAT wt) or its catalytically inactive allele (IN-HAT mut). (B) 1 μg and 2 μg of IN derived from IN-HAT wt (lanes 1 and 3, respectively), or 1 μg and 2 μg of IN derived from IN-HAT mut (lanes 2 and 4, respectively) were analyzed by Western blotting with anti-acetyl-lysine antibody (top panel) or anti-IN antibody (bottom panel). (C) 3' -end processing activity of IN derived from IN-HAT wt (lane 1: 100 ng; lane 3: 200 ng) or IN-HAT mut (lane 2: 100 ng; lane 4: 200 ng). Lane 5: DNA substrate; lane 6: DNA substrate with 40 ng of 6× His-tagged IN. (D) Strand transfer activity of IN derived from IN-HAT wt (lane 1: 100 ng; lane 3: 200 ng) or IN-HAT mut (lane 2: 100 ng; lane 4: 200 ng). Lane 5: DNA substrate; lane 6: DNA substrate with 40 ng of 6× His-tagged IN. In (C) and (D), the DNA substrate (S) and the catalytic products (P) are indicated.

To explore the effect of GCN5-mediated acetylation on the catalytic activity of IN, constitutively acetylated recombinant IN was produced by exploiting the "tethered catalysis" approach [33,34]. This method allows the production of a constitutively acetylated protein by tethering the factor of interest to the catalytic domain of a specific HAT enzyme. Based on this approach, as schematized in Figure 4A, a chimeric construct was generated where 6× His-tagged IN was fused at its C-terminal end with the HAT domain of GCN5 (amino acids 6-300). To obtain a control that cannot be acetylated, the same chimera was constructed using the inactive mutant of GCN5 Y260A/F261A. In addition, a sequence coding for Tobacco Etch Virus (TEV) protease recognition site was inserted between IN and GCN5 coding sequences to allow for the separation of the two domains. The fusion proteins expressed from the two chimeric constructs were purified, digested with TEV protease, and the acetylation levels of the resulting IN proteins analyzed by Western blotting with an anti-acetyl-lysine antibody. IN derived from the wild type GCN5 fusion scored positive for acetylation, while no significant signal was detected with IN derived from the GCN5 mutant chimera (Figure 4B, top panel, compare lanes 1 and 3 with lanes 2 and 4). In this experiment, the levels of loaded proteins were verified by incubating the same membrane with an antibody directed against IN (Figure 4B, bottom panel).

Constitutively acetylated recombinant IN and the non-acetylated control were tested *in vitro *for 3'-end processing and strand transfer activities. In the 3'-end processing reaction, recombinant IN was incubated with a [^32^P]-labeled DNA substrate (S) and the excision of 2 nucleotides evaluated by measuring the radioactive signal of the shorter product (P). In Figure 4C the comparative analysis by densitometry of the bands corresponding to the 3'-end processed template, indicated that acetylated IN (100 ng in lane 1 and 200 ng in lanes 3) was two- to three-fold more active than non-acetylated controls (lanes 2 and 4 respectively). In the strand transfer assay, a [^32^P]-labeled oligonucleotide was used as a substrate (S) and IN activity was evaluated by measuring the radioactive signal derived from the ladder of higher molecular weight products (P). Constitutively acetylated IN, at two different doses (100 ng and 200 ng), was more active than non-acetylated IN (Figure 4D, compare lanes 1 and 3 with lanes 2 and 4). This was consistent with the 3'-end processing results. Finally, densitometric analysis of the autoradiograms indicated that the two amounts of acetylated IN were five- to ten-fold more active than the corresponding non-acetylated controls.

Taken together, these results demonstrated that GCN5-mediated acetylation enhances the catalytic activity of IN *in vitro*.

### HIV-1 infectivity is reduced in GCN5 knockdown cells

**Figure 5:**
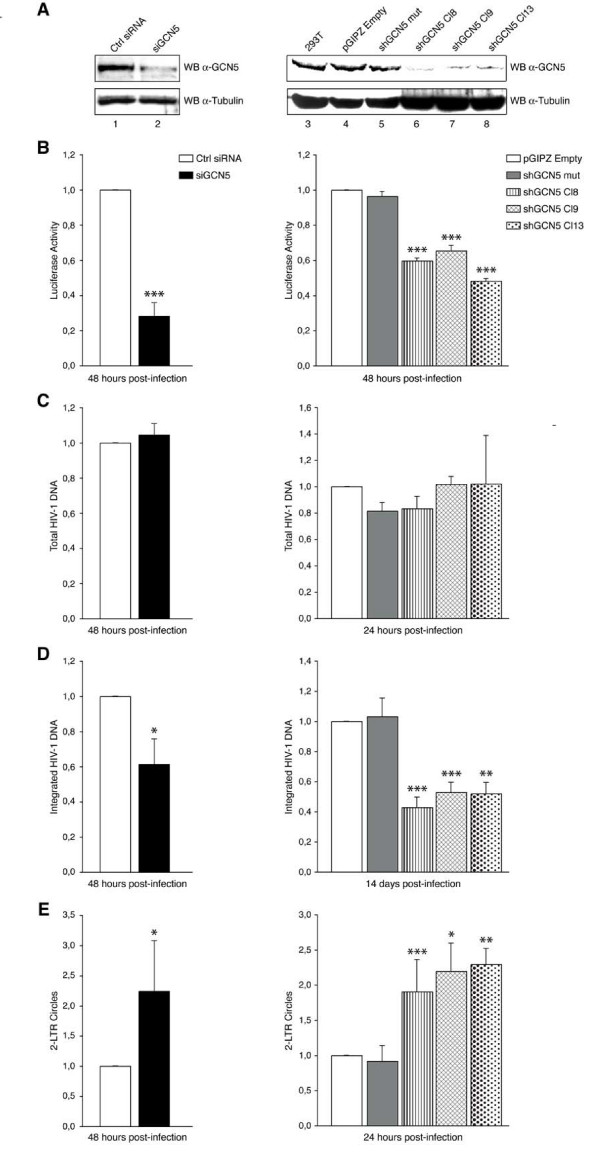
**GCN5 depletion in infected cells reduces HIV-1 integration**. (A) Left panels: extracts from siRNA-treated Hela cells analyzed by Western blotting with anti-GCN5 antibody (top) or anti-α-tubulin antibody (bottom). Lane 1: cells transfected with non-targeting siRNA (Ctrl siRNA); lane 2: cells transfected with GCN5-targeting siRNA (siGCN5). Right panels: extracts from stable GCN5 knockdown HEK 293T cell clones or control cells immunoblotted with anti-GCN5 antibody (top panel) or anti-α-tubulin antibody (bottom panel). Lane 3: untransduced HEK 293T cells; lane 4: HEK 293T cells carrying empty pGIPZ vector; lane 5: HEK 293T cells expressing mutant, non-targeting GCN5 shRNAmir; lanes 6-8: HEK 293T clones (Cl8, Cl9 and Cl13) expressing GCN5 shRNAmir. (B) siRNA-treated Hela cells (left histogram) or HEK 293T cells stably transduced with pGIPZ lentiviral vectors (right histogram) were infected with NL4.3-Luc and analyzed for luciferase activity 48 hours after infection. The histograms represent percentages of luciferase activity relative to control, non-silenced cells. Means ± SEM from three independent experiments are reported. (C-E) Total DNA extracted from siRNA-treated HeLa cells (left histograms) or HEK 293T cells stably transduced with pGIPZ lentiviral vectors (right histograms) was analyzed by RT-Q-PCR for total HIV-1 DNA (C), integrated HIV-1 DNA (D), and two-LTR circles (E). In (C-E), results are presented as percentages relative to control, non-silenced cells. Reported values are means ± SEM from three independent experiments. Statistical significance values shown in (B-E) were calculated by using the Student's two-sided *t *test. Asterisks directly above bars indicate differences between knockdown and control, non-silenced cells. ***, *P *< 0,001; **, *P *< 0,01; *, *P *< 0,05. Conversely, where asterisks are not present, values obtained did not significantly differ (*P *> 0,05) from those obtained with control, non-silenced cells.

In order to assess the physiological relevance of the IN/GCN5 interaction during HIV-1 replication cycle, viral infectivity upon GCN5 depletion in target cells was monitored. Transient knockdown of GCN5 expression was obtained in HeLa cells using a specific short interfering RNA (siRNA), while stably silenced HEK 293T cell clones were selected after transduction with a lentiviral vector (pGIPZ from Open Biosystems, Inc.) encoding a short hairpin RNA (shRNA) targeting GCN5 (GCN5 shRNAmir). As a control for the transient knockdown experiments, HeLa cells were transfected with a non-targeting siRNA (unrelated to any human genomic sequence), while stable silencing experiments were checked by using two HEK 293T polyclonal cell lines, one expressing a mismatched, non-targeting GCN5 shRNAmir (GCN5 shRNAmir mut) and the other carrying an empty pGIPZ vector. As shown in the top panels of Figure 5A, siRNA- and shRNAmir-mediated knockdown reduced GCN5 expression to a similar extent. Silenced cells were then infected with an *env*-deleted, VSV-G pseudotyped NL4.3 virus expressing the luciferase reporter gene (indicated hereafter as NL4.3-Luc), and luciferase activity was measured 48 hours after infection. As shown in Figure 5B, a two- to three-fold reduction in luciferase activity was detected in both transiently and stably silenced cells, thus indicating that knockdown of GCN5 expression in target cells reduces HIV-1 infectivity. To determine which step of viral replication was affected by GCN5 depletion, cells were collected at various time points after infection, and measurements of the different HIV-1 DNA species were performed by real time quantitative PCR (RT-Q-PCR). Total HIV-1 DNA was quantified with the use of primers annealing to the luciferase reporter gene, in order to avoid cross-reaction with the integrated pGIPZ lentiviral vectors present in stably transduced cell lines. As shown in Figure 5C, no significant alterations in total HIV-1 DNA levels were detected in cells either transiently or stably silenced, thus indicating that reverse transcription was not affected by the reduction of GCN5 expression. SiRNA-treated cells were analyzed 48 hours post-infection by Alu-LTR nested PCR to detect integrated HIV-1 DNA, while stable knockdown cell clones were processed two weeks after infection using primers specific to the luciferase gene. This was necessary in order to dilute non-integrated HIV-1 DNA and avoid cross-reaction with the integrated pGIPZ lentiviral vectors. Proviral DNA was about two-fold less in all GCN5 knockdown cells, either treated with siRNA or transduced with shRNAmir-encoding lentiviral vectors (Figure 5D). Finally, a two-fold increase in the amount of two-LTR circles was detected in both stably and transiently silenced cells (Figure 5E). Since the increase in two-LTR circles often correlates with a defect at the step of integration [35], these data are collectively consistent with reduced integration efficiency in GCN5 knockdown cells.

### Mutations at IN acetylation sites cause a defect in HIV-1 replication at the integration step

**Figure 6:**
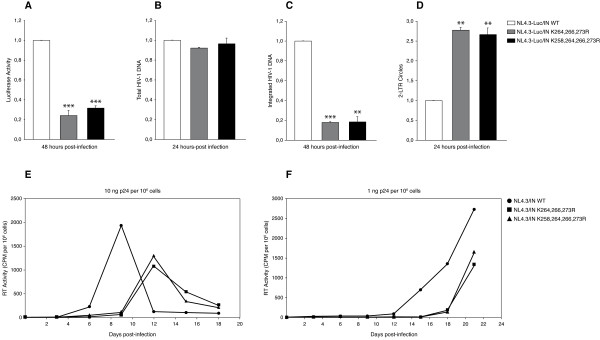
**Mutations at IN acetylation sites cause a replication defect at the step of integration**. (A) HEK 293T cells infected with NL4.3-Luc/IN WT, NL4.3-Luc/IN K264,266,273R, or NL4.3-Luc/IN K258,264,266,273R were analyzed for luciferase activity 48 hours after infection. (B-D) Total DNA extracted from HEK 293T cells infected with the same viral clones as in (A) was analyzed by RT-Q-PCR for total HIV-1 DNA at 24 hours after infection (B), integrated HIV-1 DNA at 48 hours after infection (C) and two-LTR circles at 24 hours after infection (D). In (A-D), results are presented as percentages relative to cells infected with NL4.3-Luc/IN WT virus. Reported values are means ± SEM from three independent experiments. Statistical significance values shown in (A-D) were calculated by using the Student's two-sided *t *test. Asterisks directly above bars indicate differences between cells infected with mutant viruses and cells infected with wild type virus. ***, *P *< 0,001; **, *P *< 0,01. Conversely, where asterisks are not present, values obtained did not significantly differ (*P *> 0,05) from those obtained with cells infected with wild type virus. (E) RT activity detected in the culture supernatants of CEM cells at different time points after infection with NL4.3/IN WT, NL4.3/IN K264,266,273R, or NL4.3/IN K258,264,266,273R. (F) Infections performed as in (E), using 10-fold lower viral loads.

Since the IN lysines acetylated by GCN5 partially overlap with those targeted by p300, a comparative analysis was performed to evaluate the role of these residues during the HIV-1 replication cycle. To this aim, single-round infections were performed, using *env*-deleted NL4.3-Luc viruses expressing either IN K264,266,273R (NL4.3-Luc-3mut), or IN K258,264,266,273R (NL4.3-Luc-4mut). Luciferase activity was measured 48 hours after infection, revealing an average five-fold reduction in infectivity for both mutant viruses as compared to wild type (Figure 6A). To determine which step of viral replication was affected by the lysine-to-arginine substitutions, DNA was extracted from cells at several time points after infection and the different HIV-1 DNA species were measured by RT-Q-PCR. Infection with NL4.3-Luc-3mut and 4mut, as well as with wild type virus, resulted in similar levels of total HIV-1 DNA at 24 hours post-infection (Figure 6B), indicating that reverse transcription was not affected by the amino acidic substitutions. Integrated HIV-1 DNA was quantified at 48 hours post-infection by Alu-LTR nested PCR, showing a five-fold reduction in the number of proviruses for both mutant clones with respect to wild type (Figure 6C). These data indicated decreased integration efficiency upon mutation of IN lysines targeted by acetylation. Consistently, a three-fold increase in the amount of two-LTR circles was detected at 24 hours post-infection with both NL4.3-Luc-3mut and 4mut (Figure 6D), confirming a specific defect at the step of integration and no alterations during viral nuclear import.

To investigate the role of IN acetylated lysines during HIV-1 replication in a T-cell line, two NL4.3-derived clones were generated, expressing either the triple- or quadruple-mutant IN (NL4.3-3mut and NL4.3-4mut, respectively). One million CEM T-cells were infected with the resulting viruses using two different amounts of p24 antigen (10 ng or 1 ng). Viral replication was followed by measuring HIV-1 reverse transcriptase (RT) activity in the culture supernatants every three days over a period of 21 days. As shown in Figure 6E, cells infected with the higher viral load (10 ng of p24) of wild type virus showed a peak of HIV-1 replication around day 9 post-infection. Conversely, infections with the same amounts of NL4.3-3mut and -4mut resulted in delayed peaks at day 12. Notably, at the infectivity peak, the RT amounts produced by both mutant HIV-1 clones were approximately half of that obtained with wild type virus. By using the lower viral load (1 ng of p24), the replication curve of wild type virus started to raise quite steeply around day 12 post infection, while for both mutant clones the curves started to appear at day 15. Detectable RT production was observed for both mutant viruses at day 18, thus with 6 days of delay compared to the kinetics of the wild type virus (Figure 6F). In conclusion, mutations introduced in the virus at IN acetylation sites targeted by both GCN5 and p300 (K264, K266, and K273), or additional mutation at lysine 258 specifically acetylated by GCN5 *in vitro*, determined similar decreases in viral integration and infectivity.

## Discussion

**Figure 7:**
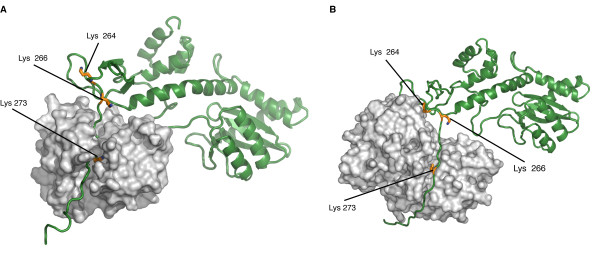
**Three-dimensional models of IN complexes with GCN5 and p300**. (A) Three-dimensional model of the IN/GCN5 complex. IN is represented in green and GCN5 in light grey. (B) Three-dimensional model of the IN/p300 complex. IN is represented in green and p300 in light grey. In (A) and (B), the three lysine residues in the C-terminal domain of IN that are acetylated by both GCN5 and p300 (Lys 264, Lys 266, and Lys 273) are shown in yellow. GCN5 and p300 are rendered as surfaces, while IN as a cartoon to highlight the C-terminal unfolded portion which inserts in the binding pockets of the two HATs.

The results presented in this study reveal that GCN5 is a novel HAT which interacts with IN. GCN5 binding to the C-terminal domain of IN leads to the acetylation of IN at lysines 258, 264, 266 and 273, located within the same region required for the two proteins to interact. We have recently demonstrated that the carboxy terminus of IN is a substrate for another cellular HAT, p300, which acetylates IN lysines at positions 264, 266, and 273 [1], a finding that was also later confirmed by Topper and coworkers [2]. Therefore, based on previous and present studies, three IN lysines (K264, K266, and K273) are acetylated by both HATs, while lysine 258 appears to be specifically targeted by GCN5. Our mapping of the HAT-interacting regions of IN based on *in vitro *binding assays is consistent with a recent report which presented two models of full-length IN complexed with GCN5 and p300 [36]. Both models predict that the IN C-terminal tail located between amino acids 271 and 288, due to its high flexibility, could easily adapt to the binding pocket of GCN5, as well as to that of p300 (Figure 7). Interestingly, lysine 273 is included in this unstructured region and is therefore expected to be the residue most prone to acetylation. In fact, since lysines 264 and 266 are located in close proximity to a sandwich of two three-stranded antiparallel β-sheets, their binding and acetylation would require a more complex unfolding of this stable secondary structure. Based on this model, we may hypothesize that IN lysine 273 is the first residue contacted and acetylated by the HAT enzyme, whether GCN5 or p300. This event might in turn induce a conformational change in the C-terminal portion of IN, which could facilitate the modification of the other two lysines. This hypothesis is also compatible with the data reported by Topper and coworkers, demonstrating a hierarchy of reactivity between the three residues modified by p300, with lysine 273 as the key site targeted for acetylation [2].

A comparative analysis, aimed at establishing the roles of the two HATs during the HIV-1 replication cycle, revealed that the mutant viruses expressing either IN K264,266,273R or IN K258,264,266,273R exhibited the same replication deficiency, specifically affecting the step of integration. These results indicated that acetylation of IN C-terminal lysines 264, 266, and 273 is required for maximal HIV-1 integration efficiency, while acetylation of lysine 258, although observed *in vitro*, does not appear to play any significant role during infection.

Proteins modified by acetylation, including viral factors, are often targeted by multiple HATs in a redundant manner. For instance, HIV-1 Tat is acetylated at lysines 50 and 51 by p300/CBP and GCN5, leading in both cases to an increased transactivation activity of the modified protein on the viral LTR promoter [27-30]. The action of two different HATs on common sites of the same substrate may be ascribed to the importance of acetylation for the functionality of the target protein. However, in the case of IN, the reduced viral integration capacity detected in GCN5 knockdown cells indicated that endogenous p300 is not able to fully compensate for the lack of GCN5 so as to completely restore HIV-1 infectivity.

The role of IN acetylation at lysines 264, 266 and 273 during the HIV-1 replication cycle has been the subject of a recent debate. Our former study showed that the replication level of a HIV-1_BRU _clone expressing a triple-mutant Flag-tagged IN (Flag-IN K264,266,273R) was severely impaired, and that the replication deficiency was specifically due to a block at the integration step [1]. In subsequent reports, the untagged triple-mutant virus showed either no replication defect [2], or a five-fold infectivity decrease in single-round infections [37]. Moreover, by using a genetic assay where integration was evaluated through the number of cell clones containing proviruses, one report [2] detailed almost half decreased integration efficiency, while the other [37] indicated a 14-fold lower residual integration rate. In the present study, we performed single- and multiple-round infections with HIV-1 clones encoding IN either mutated at the positions targeted by both GCN5 and p300 (IN K264,266,273R), or carrying an additional lysine-to-arginine substitution at the site specifically modified by GCN5 (IN K258,264,266,273R). In multiple-round replication experiments, both mutant clones showed reduced virus production and delays in the peaks of infectivity with respect to wild type. The discrepancy of these findings with the data reported by Topper *et al. *[2] might be due to the different time-courses of analyses: although working in the same experimental conditions (10 ng of p24 antigen per 1× 10^6 ^CEM cells), the detection of RT activity in the culture supernatants over a period of 21 days allowed us to monitor the peak of HIV-1 replication, while Topper and coworkers terminated the replication curve before the highest point of viral infectivity was reached (at 12 days post infection).

Moreover, consistent with Apolonia *et al. *[37], we detected a five-fold infectivity decrease in single-round replication assays performed with IN triple- and quadruple-mutant viruses. The five-fold infectivity decrease paralleled a five-fold reduction in the number of proviruses, as measured by RT-Q-PCR. Taken together, the results presented in all the different reports suggest that acetylation of IN C-terminal lysines 264, 266, and 273 represents a mechanism which, by finely regulating the integration process, contributes to determine the efficiency of HIV-1 replication.

Identification of lysines 258, 264, 266, and 273 as the targets of GCN5 activity on IN does not exclude that additional residues might be acetylated, as indicated by the residual acetylation level of the quadruple-mutant IN (Figure 1A, lane 15). Finally, IN could also be subject to different post-translational modifications, such as methylation, sumoylation, or ubiquitination [38-41], which might open up new mechanisms of modulation of IN function.

## Conclusions

This study demonstrates that, in addition to the formerly reported p300, another HAT, GCN5, acetylates the C-terminal domain of IN. Similar to p300, GCN5-mediated acetylation is required for efficient viral integration, thus reinforcing the role of this post-translational modification for HIV-1 replication.

## Methods

### Plasmids

Construction of pGEX-IN has already been described [1]. pcDNA3-HA-IN was obtained by subcloning IN sequence from pGEX-IN plasmid into pcDNA3-HA vector. pGEX-IN and pcDNA3-HA-IN deletion mutants were produced by PCR amplification of IN with primers specific to the deleted versions. pASK-IBA37-IN was constructed by subcloning IN sequence from pGEX-IN plasmid into pASK-IBA37 vector (IBA GmbH, Göttingen, DE). pFlag-IN codon optimized (c.o.) was kindly provided by A. Engelman. pASK-IBA37-IN point mutants and pFlag-IN c.o. K264,266,273R or K258,264,266,273R were obtained by PCR-based site-directed mutagenesis starting from the corresponding plasmids encoding wild type IN.

pGEX-GCN5 was a kind gift of M. Benkirane. pGEX-GCN5 deletion mutants were produced by PCR amplification of GCN5 with primers specific to the truncated forms. pcDNA3-HA-GCN5 was constructed by subcloning GCN5 sequence from pGEX-GCN5 plasmid into pcDNA3-HA vector. pcDNA3-HA-GCN5 (Y260A/F261A) [32] was obtained by PCR-based site-directed mutagenesis starting from the plasmid encoding wild type GCN5.

For production of IN-GCN5 tethered catalysis constructs, the sequence coding for the 6-300 amino acid region of GCN5 was amplified by PCR from pcDNA3-HA-GCN5 or pcDNA3-HA-GCN5 (Y260A/F261A) and cloned into a pASK-IBA37 vector in frame with c.o. IN. The sequence encoding TEV protease recognition site was inserted by PCR between IN and GCN5 cDNAs.

pGIPZ and pGIPZGCN5 lentiviral vectors were purchased from Open Biosystems (Huntsville, AL). The sequence of GCN5 shRNAmir inserted into the pGIPZGCN5 vector is as follows: 5'-CCCATTCATTCCCTGGCATTAATAGTGAAGCCACAG ATGTATTAATGCCAGGGAATGAATGGT-3'. For production of the pGIPZGCN5 mut vector, four point mutations were introduced in the shRNAmir cassette of pGIPZGCN5, obtaining the following sequence: 5'-CCCATTCA**AAGG**CTGGCA TTAATAGTGAAGCCACAGATGTATTAATGCCAG**CCTT**TGAATGGT-3', where mutated nucleotides are underlined.

The NL4.3-Luc *env*-deleted virus expressing the luciferase reporter gene was produced from the pNL4.3.Luc.R-E- molecular clone obtained from the AIDS Research and Reference Reagent Program, Division of AIDS, NIAID, NIH. IN sequence was subcloned from the molecular clone pHXB2 for construction of pNL4.3.Luc.R-E-/IN WT and pNL4.3/IN WT plasmids. The IN mutations in pNL4.3.Luc.R-E-/IN K264,266,273R, pNL4.3.Luc.R-E-/IN K258,264,266,273R and in pNL4.3/IN K264,266,273R, pNL4.3/IN K258,264,266,273R were introduced by PCR-based site-directed mutagenesis using either pNL4.3.Luc.R-E-/IN WT or pNL4.3/IN WT as template.

The envelope plasmid pMDG and the packaging plasmid pCMVΔR8.91 were kindly provided by Z. Debyser.

### *In vitro *acetylation assay

HAT assays were performed as previously described [1], with minor modifications. Briefly, GST or 6× His-tag fusion proteins were incubated with GST-GCN5 and [^14^C]-acetyl-CoA in HAT buffer (50 mM Tris-HCl, pH 8.0, 5% glycerol, 0.1 M EDTA, 50 mM KCl and 2 mM sodium butyrate) in a final volume of 30 μl for 45 min at 30°C. Acetylated proteins were visualized by phosphoimaging (Cyclone) after separation by SDS-PAGE.

### In vitro binding assay

[^35^S]-labeled IN proteins used for *in vitro *binding assays were produced from the corresponding pcDNA3-HA plasmids by using the TNT Reticulocyte Lysate System (Promega Corp., Madison, WI). Analysis of *in vitro *binding between GST fusion proteins and [^35^S]-IN or [^35^S]-IN fragments was performed as previously described [1]. Briefly, GST fusion proteins (1 μg) immobilized on agarose beads, after pre-treatment in a solution containing DNase I 0.25 U/μl and RNase H 0.25 U/μl, were incubated with 600 c.p.m. of *in vitro *translated [^35^S]-proteins in a solution containing 0.2 mg/ml ethidium bromide. Following extensive washes, the reaction mixtures were resolved by SDS-PAGE and radiolabeled proteins visualized by phosphoimaging (Cyclone).

### Recombinant proteins production and proteolytic processing

GST fusion proteins were expressed and purified from *Escherichia Coli *BL21 as already described [1].

N-terminal 6× His-tagged IN proteins were expressed in *Escherichia Coli *BL21 and purified by metal ion affinity chromatography (BD TALON Metal Affinity Resin, BD Biosciences, Palo Alto, CA) according to a previously reported protocol [42]. Proteolytic processing of IN-GCN5 chimeras was performed by incubating 20 μg of fusion protein with 30 U of TEV protease (AcTEV Protease, Invitrogen, Inc., Carlsbad, CA) in a buffer containing 50 mM Tris-HCl, pH 8.0, 0.5 mM EDTA, 0.1 M NaCl, 1 mM DTT and 10% glycerol, overnight at 4°C. 6× His-tagged IN was then recovered from the reaction mixture by adsorption on BD TALON Resin.

### Immunoprecipitation and Western blotting

For immunoprecipitation, cell pellets were lysed 36 hours after transfection in RIPA buffer (50 mM Tris-HCl, pH 7.5, 150 mM NaCl, 1% Triton X-100, 0.1% SDS, 0.5% deoxycolic acid) containing 10 mM sodium butyrate (Sigma, Inc.) and protease inhibitors (Complete Protease Inhibitor Cocktail Tablets, Roche Diagnostics). Anti-Flag M2 affinity resin or rat monoclonal anti-HA antibody were incubated overnight at 4°C with the cell lysates (2 mg for coimmunoprecipitation or 4 mg for in vivo acetylation experiments). The HA-immune complexes were precipitated by incubation with UltraLink Immobilized Protein G (Pierce Biotechnology, Inc., Rockford, IL). The precipitated complexes were then extensively washed and analyzed by Western blotting using the appropriate antibodies.

### Antibodies

The following primary antibodies were used: rabbit anti-acetylated-lysine (Cell Signaling Technology, Inc., Danvers, MA); mouse anti-Flag M2 (Sigma, Inc., St Louis, MO), either free or bound to agarose beads; rat anti-HA Clone 3F10 (Roche Diagnostics, Indianapolis, IN); mouse anti-IN 8G4, obtained from the AIDS Research and Reference Reagent Program; rabbit anti-GCN5 H-75 (Santa Cruz Biotechnology, Inc., Santa Cruz, CA) and mouse anti-α-tubulin Clone B-5-1-2 (Sigma, Inc.).

For the production of a polyclonal, anti-acetylated IN antibody, three rabbits were immunized with a peptide corresponding to amino acids 261-280 of the IN sequence, chemically acetylated at lysines 264, 266 and 273, after conjugation with Maleimide Activated mcKLH (Pierce Biotechnology, Inc.). The IgG fraction was obtained from collected sera with the use of ImmunoPure (A) IgG Purification Kit (Pierce Biotechnology, Inc.). The purified samples were then passed over a column conjugated with the unmodified IN peptide to remove the antibody cross-reacting with non-acetylated IN.

Secondary horseradish peroxidase (HRP)-conjugated antibodies against mouse or rabbit Igs were purchased by Santa Cruz Biotechnology, Inc. For Western blot analysis with anti-acetylated-lysine antibody, Biotin-SP-conjugated AffiniPure F(ab')2 Fragment Donkey Anti-Rabbit IgG (H+L) (Jackson ImmunoResearch Laboratories, Inc., West Grove, PA) and ECL Streptavidin-HRP conjugate (Amersham Biosciences Corp., Piscataway, NJ) were employed.

### Cell cultures and virus production

HeLa and HEK 293T cells were cultured in DMEM supplemented with 10% fetal calf serum (FCS), 100 U/ml penicillin and 100 μg/ml streptomycin. HEK 293T cells stably transduced with pGIPZ vectors were grown with the addition of puromycin 2 μg/ml. CEM cells were cultured in RPMI 1640 supplemented with 10% FCS, 2 mM glutamine, 100 U/ml penicillin and 100 μg/ml streptomycin.

To produce *env*-deleted, VSV-G pseudotyped NL4.3-Luc viruses, 6× 10^6 ^HEK 293T cells were transfected with 20 μg of pNL4.3.Luc.R-E- (wild-type or mutated) and 5 μg of the envelope plasmid pMDG using the PEI reagent (Sigma, Inc.). The cell culture supernatant was collected 48 h after transfection and filtered through a 0.45 μM pore size filter.

NL4.3 replication competent viruses were prepared as described for NL4.3-Luc viral clones, using 25 μg of pNL4.3 plasmid (wild-type or mutated) for transfections.

For the generation of viral vector stocks, HEK 293T cells were transfected with 10 μg of the packaging plasmid pCMVΔR8.91, 5 μg of pMDG and 20 μg of the gene transfer plasmid (pGIPZ, pGIPZGCN5, or pGIPZGCN5 mut), following the protocol used for virus production. The cell culture supernatant was collected twice, at 48 h and 72 h after transfection, filtered through a 0.45 μM pore size filter and concentrated by ultracentrifugation at 110000 × g for 2 h at 4°C.

Both viruses and viral vectors were titered by quantification of p24 antigen in cell culture supernatants with an enzyme-linked immunoabsorbent assay (Innogenetics, Gent, Belgium).

### Transient and stable knockdown of GCN5 expression

GCN5-targeting siRNA (Dharmacon Research, Boulder, CO) had the following plus-strand sequence: 5'-AACCAUGGAGCUGGUCAAUGAAA-3'. As a non-silencing control, Dharmacon ON-TARGETplus siCONTROL Non-Targeting Pool was employed.

HeLa cells, seeded in 6-well plates (1.5 × 10^6 ^cells/well), were transfected twice at a 24 h interval with 150 nM siRNA using Gene Silencer reagent as recommended by the manufacturer (Gene Therapy Systems, Inc., San Diego, CA). Cells trypsinized after 20 h were either plated for infections, or lysed for Western blot analysis.

For production of stably silenced cell lines, HEK 293T cells, seeded in 24-well plates (5 × 10^4 ^cells/well), were transduced with shRNAmir-encoding pGIPZ lentiviral vectors and grown in medium containing 2 μg/ml puromycin.

### Infectivity and IN activity assays

For single-round replication assays, siRNA-treated HeLa cells (2.5 × 10^6^/well) or HEK 293T cells (5 × 10^6^/well) were seeded in 6-well plates and incubated for 3 h, in a total volume of 500 μl, with 50 or 100 ng p24 antigen of NL4.3-Luc virus (wild type or mutated), respectively. Cells were collected 48 h after infection for measurement of luciferase activity (Luciferase Assay System, Promega Corp.).

Viral stocks used in infections for measurement of HIV-1 DNA species by RT-Q-PCR were pre-treated for 1 h at 37°C with 160 U/ml Turbo DNase (Ambion, Inc., Austin, TX).

For multiple-round infections, 1 × 10^6 ^CEM cells were incubated with 1 ng or 10 ng p24 antigen of NL4.3 virus (wild-type or mutated) in a total volume of 500 μl for 3 h. Every 3 days, supernatants were collected and viral titers determined by a ^32^P-based RT assay performed by standard procedures.

To evaluate IN catalytic activity *in vitro*, 3'-end processing and strand transfer reactions were performed with recombinant IN proteins as previously described [1].

### Real-time quantitative PCR analysis

Total DNA was extracted from HEK 293T cells with the DNeasy Tissue Kit (QIAGEN, Valencia, CA) at different time points after infection. Amplification reactions were performed with the Light Cycler 480 instrument (Roche Diagnostics). Quantification of total HIV-1 DNA was performed with a pair of primers and a fluorogenic hybridization probe annealing to the luciferase reporter gene of NL4.3-Luc viral clone. The sequences of the primers and the probe are as follows: forward primer, LucFw, 5'-GAAGAGATACGCCCTGGTTCC-3'; reverse primer, LucRev, 5'-TGTGATTTGTATTCAGCCCATATCG-3'; and probe, LucProbe, 5'-FAM-TTCATAGCTTCTGCCAACCGAACGGACA-3' - BlackBerry Quencher. Reaction mixtures contained 500 ng of total genomic DNA, 1× Light Cycler 480 Probe Master (Roche Diagnostics), 300 nM each forward and reverse primers and 200 nM probe in a total volume of 20 μl. After an initial denaturation step (95°C for 10 min), the cycling profile was 40 cycles consisting of 95°C for 30 s, 60°C for 30 s, and 72°C for 30 s. Quantifications of proviral DNA at 48 h post infection (Alu-LTR nested PCR) and of two-LTR circles were performed according to previously described protocols [43]. For detection of integrated HIV-1 DNA in HEK 293T cells transduced with pGIPZ vectors, cells were maintained in culture for two weeks and proviruses were quantified using LucFw, LucRev primers and LucProbe.

As an internal standard for normalizing the amount of cellular genomic DNA, the level of human β-globin DNA was determined in each sample using primers and fluorogenic hybridization probe that were previously described [44]. The amplification conditions included a hot start at 50°C for 2 min and 95°C for 10 min, followed by 40 cycles of denaturation at 95°C for 15 s and extension at 60°C for 1 min.

### Statistical analysis

Paired comparisons were carried out using two-tailed Student's *t*-tests, assuming equal variance between samples to determine differences at the 5% level; all data points (including outliers) were included in the analysis for significance.

## Competing interests

The authors declare that they have no competing interests.

## Authors' contributions

MT designed and performed the experiments, analyzed the data, wrote the manuscript; PV performed the experiments and analyzed the data; VL designed the experiments and analyzed the data; MIG performed the experiments and analyzed the data; CDP performed the experiments and helped in the design of the study; ADF performed the computational analysis; VT performed the computational analysis; AAll performed the experiments and analyzed the data; AAlb performed the experiments and analyzed the data; MG designed the research and analyzed the data; AC designed the research, analyzed the data and wrote the manuscript
